# Expression of CDX2 in gastric cardia adenocarcinoma and its correlation with H. pylori and cell proliferation

**DOI:** 10.18632/oncotarget.10362

**Published:** 2016-07-01

**Authors:** Ying Zhang, Hu Wang, Chao Bi, Yinping Xiao, Zhaoyong Liu

**Affiliations:** ^1^ Department of Pathology, Shantou University Medical College, Shantou, Guangdong Province, China; ^2^ Department of Orthopaedics, First Affiliated Hospital of Shantou University Medical College, Shantou, Guangdong, China

**Keywords:** CDX2, Helicobacter pylori, gastric cardia cancer, cell proliferation

## Abstract

**Background:**

Gastric cardia cancer (GCC) is located in the distal stomach, and strongly correlates with atrophic gastritis and *Helicobacter pylori(H.pylori)* infection. Caudal-related homeobox transcription factor 2 (CDX2) is homeobox gene encoding an intestine-specific transcription factor usually expressed in the intestinal epithelium cells. However, in several recent published papers, CDX2 was found to be aberrantly expressed in gastric, thyroid and ovarian cancer.

**Results:**

Higher expression of CDX2 was found in GCC tissues in comparison with non-malignant cardia mucosa (p<0.05). Moreover, immunohistochemical analysis demonstrated that CDX2 expression correlated with lymphatic metastasis. In addition, we found that CDX2 expression progressively increased with the level of *H. pylori* infection (p<0.05), and also correlated with cell proliferation, based on Ki67 staining.

**Methods:**

To investigate the relationship between CDX2, cell proliferation and *H. pylori* infection, we detected CDX2, Ki62 and *H.pylori* expression in 83 non-malignant gastric cardia mucosacases and 60 GCC specimens in the Chaoshan area, a high-risk region for esophageal and gastric cardia cancer.

**Conclusion:**

These findings provide pathological evidence that *H. pylori* infectionis a driving force of gastric cardia carcinogenesis by upregulating CDX2 and inducing inflammation. These results provide new pathological evidence that *H. pylori* infection induces GCC tumorigenesis.

## INTRODUCTION

Gastric cardia cancer (GCC) occurs at the top of the stomach, near the junction of the esophagus, and is one of the leading causes of cancer-related death worldwide. There are two distinct types of GCC, one type being located in the more distal stomach as a consequence of atrophic gastritis, and the other type resembling esophageal adenocarcinoma, which is likely to be a consequence of short-segment gastro-esophageal reflux disease [1, 2]. Earlier studies demonstrate the incidence of the gastric cardia cancer to be 4% to 10% per year [3]. However, the mechanisms for the development of GCC have not been well-demonstrated up to now. The Chaoshan region has one of the highest incidences of esophageal cancer and GCC, with age-standardized incidence rates for GCC in the Chaoshan area being 34.81/100,000 [4], which suggests unique environmental/genetic factors involved in GCC pathogenesis.

*Helicobac*t*e*r *pylori (H. pylori)* gastric infection has been reported to be the main risk factor in gastric cardia cancer [5]. *H. pylori* is a gram-negative bacterium that is adapted to survive in the human stomach microenvironment. It is estimated that *H. pylori* infects more than half of the human population [6]. *H. pylori* can activate the host immune response, thus leading to chronic gastritis and ulcers. A small number of patients may develop gastric cancer, after long-term *H. pylori* infection, through mechanisms demonstrated in previous reports [7–11]. One mechanism involves *H. pylori*-mediated generation offree radicals to lead to increasesin gene mutations. Another mechanism involves *H. pylori*-induced pro-inflammatory genes, such as interleukin-6 and TNF-α, which are highly expressed in *H. pylori*-infected tissues [12]. These factors can alter gastric epithelial cell adhesion and lead to the dispersion and migration of mutated epithelial cells [13]. However, other mechanisms need to be explored.

Caudal-related homeobox transcription factor 2 (CDX2) is an intestinal transcription factor that has been reported toregulate cell differentiation, proliferation and apoptosis in normal cells [14]. Previous studies have shown that CDX2 expression is limited not only to normal intestinal cells, but also is expressed in gastric carcinoma, colorectal cancer, thyroid cancer, ovarian cancer, urinary bladder carcinoma and prostate adenocarcinoma [15–19]. Moreover, *H. pylori* infection induces abnormal expression of CDX2 in the gastric mucosa [20], suggesting a possible role for CDX2 in gastric cardia. However, the expression of CDX2 in gastric cardia and its correlation with *H. pylori* infection has not been clearly demonstrated. In this paper, we use immunohistochemistry and Giemsa staining to detect the of and determine the correlation between CDX2, Ki67 and *H. pylori* infection in a large cohort of GCC patients and non-malignant cardia mucosa to explore new mechanisms by which *H. Pylori* infection promotes GCC carcinogenesis.

## RESULTS

### CDX2 expression in gastric cardia specimens

**Figure 1:**
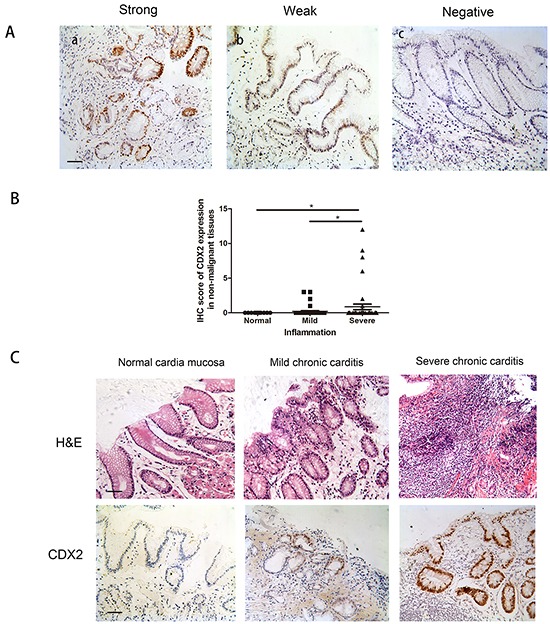
CDX2 expression in non-malignant gastric cardia tissues **A.** Variable levels of CDX2 were detected by immunohistochemistry in a small number of non-malignant gastric cardia tissues. Analysis was based on nuclear staining. Patient samples were categorized into strong (a), weak (b) and negative (c) (IHC stain, scale bar, 50 μm). **B.** Boxplot shows that the IHC score for CDX2, at different inflammation levels, shows that CDX2 expression was significantly higher in the severe inflammation cases involving normal tissues. **C.** Gastric cardia epithelial cases were stained with hematoxylin and eosin (H&E) and immunohistochemistry. Variable expression levels of CDX2 were detected for different severities of inflammation (IHC stain, scale bar, 50 μm) (*p<0.05).

To identify the expression of CDX2 in non-malignant gastric cardia and GCC specimens, we performed CDX2 immunohistochemical staining in a cohort of patient samples. CDX2 expression was mostly found in cell nuclei, and the staining intensity was scored as strong, weak and negative (Figure 1A). Of the 83 non-malignantgastric cardiatissues examined, there was no detectable CDX2 in 7 normal gastric cardia cases. In the remaining 76 gastric carditis cases, CDX2 was negative in 66 cases and positive in 10 cases (13.2%), including 4 CDX2-strong cases and 6 CDX2-weak cases. The immunohistochemical resultsshowed that inflamed epithelia had various expression levels of CDX2 in non-malignant gastric cardia mucosa (Figure 1B). Normal epithelia without inflammation had the no detectable CDX2, but negative/weak CDX2 staining was observed in epithelial cells accompanied by mild to severe inflammation (Figure 1C) (Table 1).

**Table 1:** Correlation between CDX2 protein expression and clinicopathological parameters in 76 gastric carditis cases

Clinical parameter		Case	CDX2 expression		*P*-value
			Negative	Positive	
**Age**	<54	35	32	3	
	≥54	41	34	7	*p*=0.326
**Gender**	Male	43	37	6	
	Female	33	29	4	*p*=1.000
**Inflammation**	+	30	30	0	
	++	20	16	4	
	+++	26	20	6	*p*=0.023*

* p<0.05

**Figure 2:**
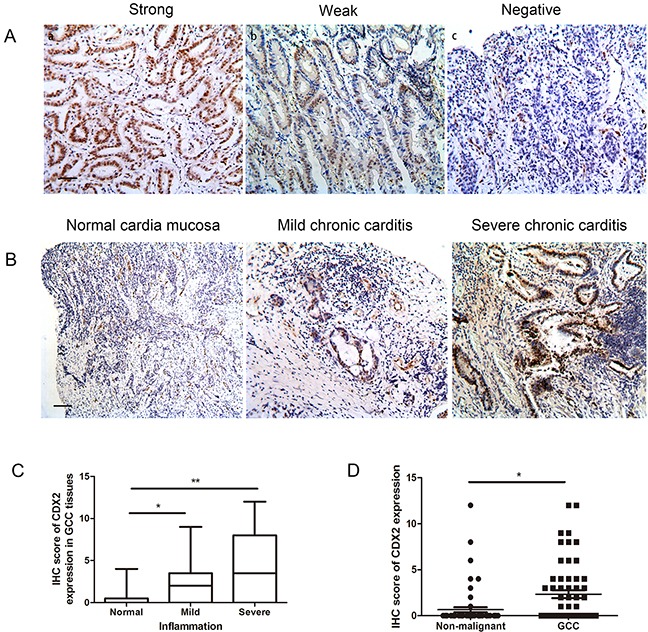
CDX2 expression in GCC tissues **A.** Variablelevels of CDX2 were detected by IHC in half of the GCC tissues. Patient samples were categorized into strong (a), weak (b) and negative (c) (IHC stain, scale bar, 50 μm). **B.** Variable expression levels of CDX2 were detected at different severities of inflammation (IHC stain, scale bar, 100 μm). **C.** Boxplot shows that IHC scoresfor CDX2 were significantly higher in the severe GCC inflammation cases than the non-inflammation cases. **D.** Boxplot shows that CDX2 expression was higher in the GCC cases compared to the non-malignant cases (*p<0.05, **p<0.01).

In 60 GCC cases, CDX2 expression was detectable in half of the cases 32/60 (53.3%), with 17/60 (18.3%) cases considered as CDX2-strong staining, and 15/60 (25.0%) cases considered as CDX2-weak staining (Figure 2A). Expression of CDX2 in the remaining 28 cases was negative. Moreover, the strong CDX2 staining was found in GCC cases with severe inflammation, whereas the weak or negative CDX2 staining was detected in the non-inflamed GCC samples (Figure 2B and 2C) (Table 2). Expression of CDX2 was significantly higher in GCC tissues than in non-malignant gastric cardia (Figure 2D). These results led us to hypothesize that CDX2 activation is a key mediatorof gastric cardia epithelial inflammation.

**Table 2:** Correlation between CDX2 protein expression and clinicopathological parameters in 60 GCC samples

Clinical parameter		Case	CDX2 expression		*P*-value
			Negative	Positive	
**Age**	≤62	29	13	16	
	>62	31	15	16	*p*=0.802
**Gender**	Male	52	24	28	
	Female	8	4	4	*p*=1.000
**Differentiation**	High	2	0	2	
	Mild	28	11	17	
	poor	30	17	13	*p=*0.173
**Invasion**	T1-T2	40	16	24	
	T3-T4	20	12	8	*p=*0.176
**Lymph node**	Yes	37	22	15	
**metastasis**	No	23	6	17	*p=*0.017*
**Inflammation**	+	15	12	3	
	++	25	10	15	
	+++	20	6	14	*p*=0.010*

* p<0.05

### Clinical significance of CDX2 expression in GCC

We then assessed whether CDX2 expression correlated with clinical parameters, such as age, gender and inflammation stage in the non-malignant gastric cardia cohort. As shown in Table 1, CDX2 expression correlated with the severity of inflammation in non-malignant gastric cardia patients (p=0.023, chi-square).

We then examined the correlation between CDX2 expression and clinical and pathological parameters, such as gender, size of the tumor, lymph node metastasis, histological grade, depth of tumor invasion and metastasis in the GCC patient samples (Table 2). GCC patients with strong CDX2 expression had a higher incidence of lymphatic metastasis and severity of inflammation (p=0.017 and p=0.010, respectively). There was no significant correlation between the expression of CDX2 andother clinical and pathological parameters in GCC. To further detect the correlation between CDX2 expression and lymph node metastasis, we divided the GCC patients into several subgroups, based on the site and number of metastatic lymphatic nodes. However, we did not find a significant correlation between either the number or site of lymph node metastases and CDX2 expression (Supplementary Table S1).

### Correlation between CDX2 expression and *H. pylori* infection

**Figure 3:**
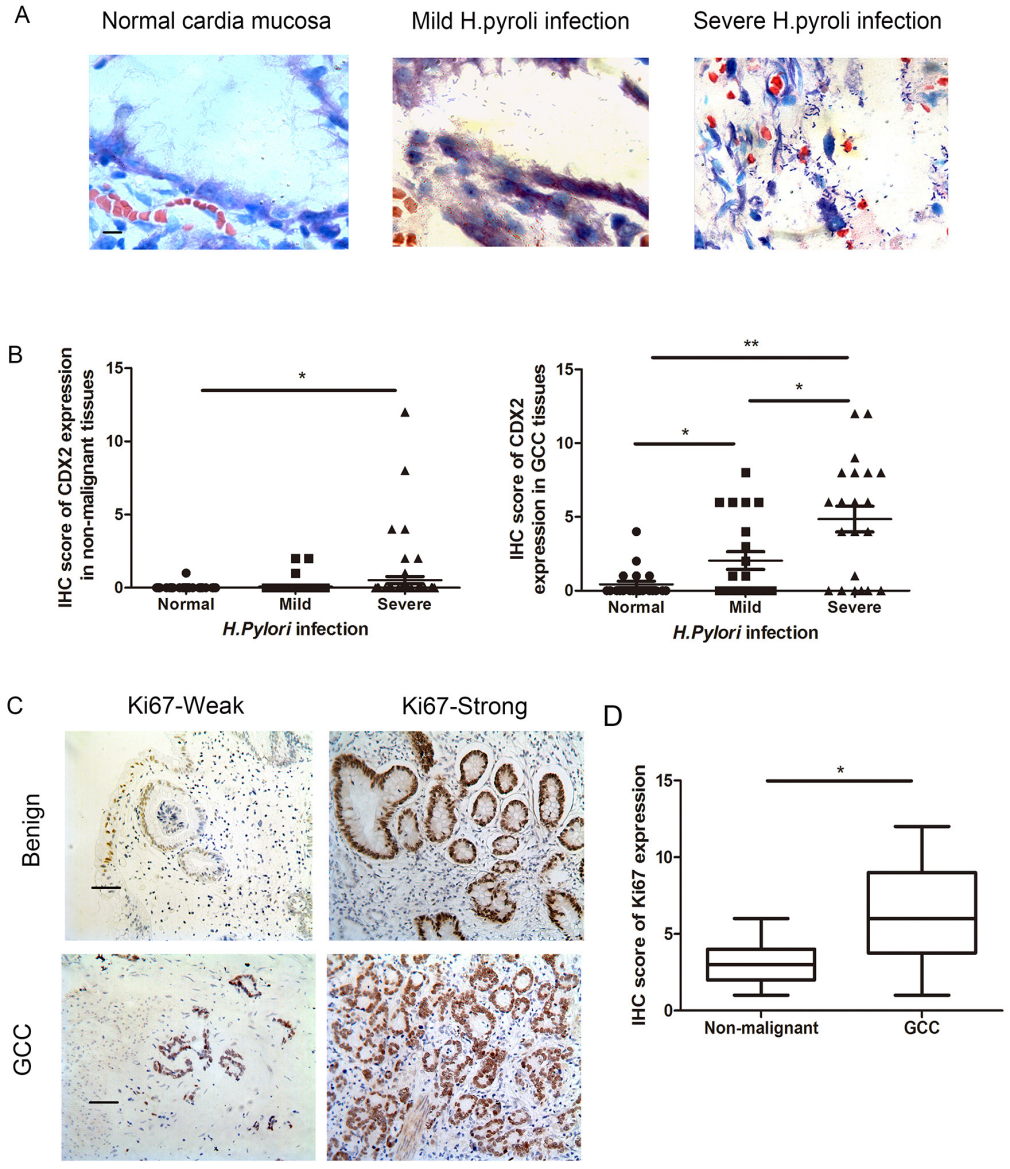
H. pylori infection and Ki67 expression in patient samples **A.** *H. pylori* infection was identified by Giemsa staining and the bacterial density was graded according to the Updated Sydney System. (Giemsa stain, scale bar, 5 μm). **B.** Boxplot shows that IHC scoresfor CDX2 were significantly higher in the severe *H. pylori* infection cases than the cases with no or mild *H. pylori* infection in both non-malignant gastric cardia and GCC tissues. **C.** IHC staining of Ki67 in non-malignant gastric cardia and GCC tissues (IHC stain, scale bar, 50 μm). All cases were divided into weak and strong subgroups. **D.** Boxplot shows that the expression of Ki67 is higher in GCC compared to the non-malignant cardia cases (IHC stain, scale bar, 50 μm) (*p<0.05, **p<0.01).

*H. pylori* infection has been shown to induce CDX2 expression in gastric cell lines [11]. Keeping this in mind, we hypothesized that CDX2 correlates with the severity of *H. pylori* infection in gastric cardia tissues. Thus, we determined the extent of *H. pylori* infection by Giemsa staining in the same cohort of non-malignant gastric cardia and GCC specimens. *H. pylori* was found in the mucous and gland epithelium in gastric carditis specimens and GCC nest (Figure 3A). The severity of *H. pylori* infection was divided into normal, mild and severe according to the Sydney system. The expression of CDX2 paralleled the severity of *H. pylori* infection from normal to severe infection in non-malignant gastric cardia and GCC patient cohort (Figure 3B). The cases with severe *H. pylori* infectionhadhigher CDX2 expression compared to cases with normal or mild *H. pylori* infection, suggesting a potential correlation with *H. pylori* infection and CDX2 expression in the gastric cardia cells.

### Correlation between CDX2 expression and cell proliferation in GCC patients

To identify the correlation between CDX2 expression and cell proliferation, we quantified expression of Ki67 in the same cohort of patient samples (Table 3). Ki67 is abiomarkerfor proliferating cells. Immunohistochemical staining detected Ki67 in all non-malignant gastric cardia tissues and GCC patient samples (Figure 3C). All GCC patients' samples were divided into Ki67-weak (n= 29) and Ki67-strong (n= 31) subgroups and non-malignant samples were categorized into Ki67-weak(n= 43) and Ki67-strong(n= 40) subgroups.

**Table 3:** Correlation between CDX2 and Ki67 in non-malignant and GCC patient samples

Ki67 expression	CDX2 expression by IHC in non-malignance samples			*P*-value
	Negative	Weak	Strong	
**Weak**	37	4	2	
**Strong**	36	2	2	p=0.754

Ki67 expression	CDX2 expression by IHC in GCC			*P*-value
	Negative	Weak	Strong	
**Weak**	18	7	4	
**Strong**	10	8	13	p=0.031*

Expression of Ki67 was higher in GCC than innon-malignant gastric cardia epithelial tissues (Figure 3D). Moreover, in GCCsamples, increased Ki67 staining in gastric cardia tissues correlated with increased CDX2 expression. The strongest immunostaining of Ki67 was observed in tumors with the highest expression level of CDX2.

## DISCUSSION

In this study, we used a cohort of gastric cardia tissues to detect the correlation between CDX2 and *H. pylori* infection, as well as inflammation and cell proliferation. We found that CDX2 is detectable in most GCC tissues and only a small set of non-malignant gastric cardia tissues. CDX2 expression is significantly higher in GCC tissues compared to non-malignant gastric cardia mucosa samples, indicating that CDX2 is up-regulated in the GCC tumorigenesis. In addition, CDX2 expression significantly correlates with the severity of inflammation in benign cardia samples and lymphatic metastasis in GCC samples, as well as the level of cell proliferation, suggesting that *H.pylori* infection may induce CDX2 expression in GCC cells, providing pathological evidence that *H.pylori* infection promotes GCC carcinogenesis.

Gastric cardia is a narrow circular zone between the esophagus and stomach, which also involves a transition from esophageal squamous epithelia to gastric mucosa. The unique location results in GCC involving both the stomach and the esophagus. Previous reports show that GCC has different characteristics from adenocarcinomas arising in the remainder of the stomach, including various risk factors and clinicopathologic characteristics, as well as alterations in gene expression [22–25]. However, most gastric cancer studies do not distinguish GCC from gastric non-cardia cancer.

CDX2 is a transcription factor that induces the transcription of target genes associated with intestinal epithelial differentiation. In normal tissue cells, CDX2 performs various important biological functions, such as cell differentiation, cell growth and cell death, especially in intestinal epithelial cells [18]. Recent studies have revealed that CDX2 is aberrantly expressed in gastric cancer, colorectal cancer, thyroid cancer, ovarian cancer, endometrial adenocarcinoma, bladder and prostate adenocarcinoma [26, 27].

*H.pylori*, a gram-negative spiral bacteria, is the most common pathogen and affects two of three populationsin the world [28]. *H.pylori* infectionmay lead to changes in the stomach environment and causeseveral diseases, such as atrophic gastritis, intestinal metaplasia, and gastric cancer. Complex mechanisms involve the induction of the major virulence factors cytotoxin-associated gene A (CagA) and vacuolating cytotoxin A (VacA) [29], which can damage the gastric mucosa and cause inflammation and cell death. The inflammatory response induced by *H. pylori* infection leads G cells to secrete the hormone gastrin, which stimulates more acid to destroy the stomach mucosa [30]. Most studies have demonstrated *H. pylori* is an important risk factor of non-cardia gastric adenocarcinoma. However, in our previous paper, we performed a combined analysis of a large cohort of GCC patient samples, from the Chaoshan area, to additionally demonstrate an association between *H. pylori* infection and risk of cardia gastric cancer, [3]. In Chaoshan, 81.5% of GCC tumors are infected with *H. pylori*, which is significantly higher than the 59% incidence of tumor infection in GCC patients in other parts of China [31], suggesting *H. pylori* infection correlates with the risk of cardia gastric cancer. However, this conclusion is not without controversy due to some studies describing an inverse relationship between H. pylori infection and gastric cardia cancer, especially in Western countries [32, 33].

The correlation between *H. pylori* and CDX2 expression has been demonstrated in several published papers, but conclusionshave been contradictory [34–49]. In *in-vivo* studies, researchers have demonstrated that CDX2 expression is significantly higher in *H. pylori*-positive cases than H. pylori-negative intestinal metaplasia patients, whereas others found that CDX2 signaling is much higher in *H. pylori*-negative patients than positive patients [34, 35]. More convincing evidence has been obtained in a 6-year follow-up study, where researchers found that CDX2 expression in the antrum is lower after eradication of *H. pylori* infection [36–40]. However, the expression of CDX2 in gastric epithelial cells did not disappear after eradication of *H. pylori* in other patient cohorts [41–46]. The difference may relateto the length of follow-up, as well as unique patient characteristics. Another potential reason is intestinal metaplasia degradation is a long-term process and that inflammation could continue even after *H. pylori* eradication. In invitro experiments, *H. pylori* infection induces CDX2 expression in the AGS gastric carcinoma cell line, but not in other cell lines [47]. All the above results indicate that *H. pylori* infection induces CDX2 expression in gastric cells [48, 49].

## MATERIALS AND METHODS

### ESCC tumor samples

We collected 60 consecutive gastric cardiacarcinoma samples and 83 non-malignant gastric cardia mucosa at the First Affiliated Hospital of Shantou between 2005 and 2012. All patients underwent potentially curative surgery without preoperative chemotherapy or radiotherapy. For each patient, tumor-surrounding non-malignant tissue (within 2 cm of the tumor) and distant non-malignant tissue (more than 5 cm away from tumor) were also analyzed. In the GCC cohort, 52 were men and 8 were women; the age range was 43-78 years, with a median of 62 years. In the non-malignant gastric cardia cohort,49 were men and 34 were women; the age was 22-77 years, with a median of 54 years. This study was approved by the ethical review committees of the Medical College of Shantou University. All participants involved in our study gave informed consent.

### Immunohistochemistry (IHC)

IHC staining was performed using the Envision Labeled Peroxidase System (Dako, Carpinteria, CA). Consecutive 4 μm-thick slices from each sample were deparaffinized in dimethyl benzene, rehydrated through a graded ethanol series and incubated with fresh 3% H_2_O_2_ for 10 min to quench endogenous peroxidase activity. After a rinse in phosphate-buffered saline (PBS), antigen retrieval was performed by microwave heating. Following incubation in 10 mmol/l citrate buffer (pH 6.0) for 20min, sections were incubated with primary antibodies for CDX2(Cell Signaling, 1:100), and Ki67 (Cell Signaling, 1:1000) at 4°C overnight. After washing, the corresponding secondary antibody (DAKO) was added for incubation at 37°C for 30 min before reaction with diaminobenzidine and counterstaining with haematoxylin. Images were captured using a Leica IM50 microscope (Imagic Bildverarbeitung AG, Wetzlar, Germany) at ×400. IHC staining was examined by two pathologists who were blinded to the clinical outcome, and a high degree of concordance between two pathologists was indicated by an inter-rater agreementkappa value of 0.903.

Scoring for CDX2and Ki67 immunostaining was performed similarly. Thus, the percentage of positive cells in each case was categorized as: 0 (<5% positive cells), 1+ (6% to 25%), 2+ (26% to 50%), 3+ (51% to 75%) or 4+ (> 75%). Staining intensity was categorized as 0 to 3+. Final scores were based on the multiplication of the percentage and intensity scores, and ranged from 0 to 12. Tumors were considered weak when they were assigned a score of 0 to 4, whereas tumors were considered strong if they had a final score of ≥6.

### Chronic inflammation grading

Inflammation ingastric cardia specimens was assessed as normal, mild and severe according to the updated Sydney System [21]. Normal gastric mucosa contains only individual (0-5)scattered inflammatory cells (mononuclear cells) in the lamina propria. However, any increase indicates chronic gastritis. Mild inflammation involving gastric mucosal mononuclear leukocytes in the lamina propria was viewed as a maximum of 5 to 30 lymphocytes, plasma cells and macrophages per highpower (×40 objective) microscopic field or, by another approach, 5 to 30 lymphocytes or plasma cells between foveolae (the area in which chronic inflammatory cells are most often found. More than 30 inflammatory cells per fieldwas considered as severe inflammation.

### Giemsa staining

For the study, 4 μm-thick sections were deparaffinized in xylene and rehydrated in a descending series of ethanol solutions, and then incubated with 0.5% hydrochloric acid alcohol for 10 min. After washing in distilled water, slides were immersed in Giemsa stain preheated to 58°C for 4 min, and immediately washed in distilled water. An additional wash in distilled water was performed after treating with 1% glacial acetic acid for 3 s, and then the slides were placed into a 52°C incubator for 30 min. Sections were dehydrated, cleared in xylene, and mounted. The *H. pylori* level was also determined according to the updated Sydney System, which categorizes infection into normal, mild and severe infection subgroups [21].

### Statistical analysis

Statistical analysis was performed with SPSS15.0 software. The correlation between CDX2 and other clinical parameters was evaluated using the chi-square or Student's t-test. A value of p<0.05 was considered as statistically significant.

## CONCLUSION

In this paper, we detect CDX2 expression in GCC, gastric carditis and normal gastric cardia tissue, and demonstrate a correlation with *H. pylori* infection, as well as cell proliferation. We find that CDX2, a specific gastrointestinally expressed protein, plays an important role in GCC carcinogenesis. CDX2 significantly correlates with *H. pylori* infection in gastric cardia cells. Our findings provides a new approach to the pathogenesis of cardiac tumors in the further.

## SUPPLEMENTARY TABLES


