# Supplementary material for: Expression of CDX2 in gastric cardia adenocarcinoma and its correlation with H. pylori and cell proliferation
Source: Oncotarget. 2016 Jul 1;7(34):54973–82. doi: 10.18632/oncotarget.10362 (PMC5342395; doi:10.18632/oncotarget.10362)
Supplement: Supplementary file 1 [file oncotarget-07-54973-s001.pdf]

Expression of CDX2 in gastriccardia adenocarcinoma and its correlation with H. pylori and cellproliferation

SUPPLEMENTARY TABLE

Supplementary Table S1: Correlation between CDX2 protein expression and lymph node metastasis

Lymph node metastasis		Case	CDX2 expression		*P*-value
			Negative 22	Positive 15	
**Numbers**	≤3	10	5	5	
	>3	27	17	10	p=0.7076
**Sites**	Esophageal	5	3	2	
	GCC	8	4	2	
	Subcarinal	10	7	3	
	Other	14	6	8	p=0.203
